# Lipid Profiling and Stable Isotopic Data Analysis for Differentiation of Extra Virgin Olive Oils Based on Their Origin

**DOI:** 10.3390/molecules25010004

**Published:** 2019-12-18

**Authors:** Igor Lukić, Alessio Da Ros, Graziano Guella, Federica Camin, Domenico Masuero, Nadia Mulinacci, Urska Vrhovsek, Fulvio Mattivi

**Affiliations:** 1 Institute of Agriculture and Tourism, Karla Huguesa 8, 52440 Poreč, Croatia; igor@iptpo.hr; 2 Fondazione Edmund Mach, Research and Innovation Centre, Via E. Mach 1, 38010 San Michele all’Adige, Italy; darosalessio92@gmail.com (A.D.R.); federica.camin@unitn.it (F.C.); domenico.masuero@fmach.it (D.M.); 3 Department of Physics, University of Trento, Via Sommarive 14, 38123 Povo Trento, Italy; graziano.guella@unitn.it; 4 NEUROFARBA, Pharmaceutical and Nutraceutical Division, University of Florence, Via Ugo Schiff 6, 50019 Sesto Fiorentino, Italy; nadia.mulinacci@unifi.it

**Keywords:** extra virgin olive oil, lipids, LC-MS/MS, NMR, IRMS, PDO

## Abstract

To differentiate extra virgin olive oils (EVOO) according to the origin of purchase, such as monocultivar Italian EVOO with protected denomination of origin (PDO) and commercially-blended EVOO purchased in supermarkets, a number of samples was subjected to the analysis of various lipid species by liquid chromatography/mass spectrometry (LC-ESI-MS/MS, LC-ESI-IT-MS) and proton nuclear magnetic resonance analysis (^1^H-NMR). Many putative chemical markers were extracted as differentiators by uni- and multivariate statistical analysis. Commercially-blended EVOO contained higher concentrations of the majority of minor lipids, including free fatty acids, their alkyl (methyl and ethyl) esters, monoglycerides, and diglycerides, which may be indicative of a higher degree of triglyceride lipolysis in these than in monocultivar PDO EVOO. Triterpenoids and particular TAG species were also found in higher proportions in the samples from the commercially-blended EVOO class, suggesting a possible influence of factors such as the cultivar and geographical origin. The largest differences between the classes were determined for the concentrations of uvaol and oleanolic acid. The results of the analysis by isotopic ratio mass spectrometry (IRMS) were reasonably consistent with the information about the geographical origin declared on the labels of the investigated EVOOs, showing considerable variability, which possibly also contributed to the differences in lipid composition observed between the two investigated classes of EVOO.

## 1. Introduction

Extra virgin olive oil (EVOO) is appreciated among consumers because of its specific flavor and nutritional properties. Due to its economic importance, EVOO is among the most common commodities subject to fraud and mislabeling, and for this reason it is protected by regulation. The international trade standard issued by the International Olive Council (IOC) [1] and the corresponding umbrella regulation in the European Union (EU) [2] include a set of analytical and sensory methods to test and confirm the quality grade and authenticity of olive oil. In EU, EVOO can be additionally protected by protected denomination of origin (PDO) [3]. PDO EVOOs are produced according to a set of specific rules set by the holder of a designation in a specification document, governing aspects such as olive cultivars used, cultivation, harvest and processing conditions, and oil physico-chemical and sensory characteristics.

In our recent case study conducted on the Italian market it has been shown that oils labelled by the highest quality category grade (EVOO), despite meeting basic regulatory requirements, can differ significantly in qualitative terms [4]. Monocultivar PDO EVOOs purchased on family farms were found to be superior to those offered at the same time in supermarkets with respect to their volatile profiles and sensory quality. Such large heterogeneity within the EVOO category can certainly influence and distort consumers’ perception of EVOO quality and in a way discredit the reputation of EVOO in general. Having in mind that the heterogeneity of oils within the EVOO category with respect to origin of purchase is certainly among the less studied topics in the ever-growing scientific area of olive oil traceability and quality, more studies are needed to find reliable chemical markers able to discriminate EVOO based on this criterion. Such findings would significantly contribute in clarifying the interrelationship between EVOO origin, overall quality and price, and would as well provide a basis for designing additional measures of protection of the PDO EVOO class in general, which is most likely target of fraud by mislabeling due to its large economic importance [5].

Triglycerides (triacylglicerols—TAGs), which are basically esters of glycerol and fatty acids (FAs), are the main neutral lipid component of olive oil (ca. 98%) [6]. Olive oil TAGs contain primarily oleic (C18:1), palmitic (C16:0), linoleic (C18:2*n*−6), stearic (C18:0), palmitoleic (C16:1), and linolenic (C18:3*n*−3) acids, while others occur in minor amounts. Monounsaturated FAs (MUFAs) and essential polyunsaturated FAs (PUFAs) are among the most important nutritional elements of EVOO. The consumption of MUFAs has been associated with decrease of several cardiovascular risk factors [7], while EVOO linoleic and linolenic acids are an important source of essential FAs in human nutrition [8]. The percentage of TAGs with equivalent carbon number 42 (ECN 42) in total TAGs, where ECN is the sum of the number of carbon atoms in three constituent FAs in TAG molecule subtracted by 2 × total of double bonds, can be used as a marker for the detection of the presence of seed oils in olive oil [2]. In combination with other olive oil constituents, TAGs have been successfully utilized as markers of varietal [9,10,11] and geographical origin of EVOO [11]. In virgin olive oils, diglycerides (DAGs) are present in a range of 1% to 3% in the form of 1,2- and 1,3-isomers, whose ratio has been used as a marker of olive oil “freshness” [12]. The percentage of total free FAs (FFAs or acidity) is one of the parameters which is evaluated for the purposes of olive oil quality categorization [1,2]. The composition of olive oil total FA (the sum of those bound with glycerol in TAGs and the FFA forms) is usually determined by gas chromatography with flame-ionization or mass spectral detection after TAG hydrolysis and methylation [2] and may provide important information about olive oil nutritional quality (level of FA unsaturation) and purity. In the last years, proton nuclear magnetic resonance (^1^H-NMR) spectral analysis of olive oil is often used to determine not only the % molar ratio of fatty acyl chains in TAG but also the relative amount of DAGs and, eventually, to detect the presence of peroxidized acyl chains in TAGs [13]. Several results have also proved the usefulness of FA composition and distribution on the glycerol moiety for the establishment of EVOO cultivar or geographical origin [14,15,16]. The concentration of alkyl esters of free FAs (FAAE), specifically ethyl esters, is included among the criteria for olive oil quality grade evaluation [1,2]. These markers originate mostly from inappropriate handling of olive fruit and oil [17,18] so their concentrations may be used to detect fraudulent mixtures of EVOO with lower quality oils, including deodorized ones.

It is general opinion that the standard profiling of TAGs, DAGs, and total FFAs has limited discriminative power to differentiate olive oils according to various criteria [19]. However, it was assumed that recent analytical developments and novel sensitive methods could be able to provide new, more specific data with more information on olive oil lipids that could be useful for EVOO differentiation.

In this work, we applied a multi-methodological approach based on several potent analytical techniques in order to find new reliable markers able to discriminate EVOO based on the origin of purchase. A method was developed based on liquid chromatography with triple quadrupole mass spectrometric detection (LC-ESI-MS/MS) for the simultaneous quantification of minor lipids, including the profiling of FFAs, which, to our knowledge, has been studied rather scarcely. As well, the method provided a more detailed composition of FA alkyl esters occurring in olive oil in relation to previous studies, supplemented by particular MAGs and triterpenoids. On the other hand, liquid chromatography with quadrupole ion-trap mass spectrometry (LC-ESI-IT-MS) was utilized for the profiling of major TAGs, but also aimed to the targeted detection of particular trace TAG species, such as C50:4, C50:3, and C56:3, as well as those containing FAs with odd number of carbon atoms, which, to our knowledge, have not been studied extensively until now. In order to provide more detailed and yet complementary data to those obtained by the other techniques mentioned above, all the samples were subjected to ^1^H-NMR quantitative analysis to obtain both the distribution among lipid species (TAG/DAG/FFA) and a reliable distribution of acyl chains unsaturation (saturated FA (SFA)/MUFA/PUFA). The latter parameter also allowed establishing the unsaturation index (UI) and the iodine value (IV), two useful chemical parameters in EVOO quality control.

Besides quality in general, the most important aspect that defines a given PDO and drives the consumers’ preferences towards this class of EVOO is in fact the authenticity of its geographical origin. One of the most useful techniques to prove and authenticate the geographical origin of EVOO is stable isotope ratio analysis by isotope ratio mass spectrometry (IRMS) [20]. For example, the stable isotope ratio of C (^13^C/^12^C, expressed as *δ*^13^C) of palmitic, oleic, and linoleic FAs was successful in differentiating olive oils according to the country or region of provenience [20,21]. Both the *δ*^13^C and ^18^O/^16^O (*δ*^18^O) ratios determined for bulk olive oils from various countries were found to change according to the latitude, the distance from the sea and the environmental conditions during growing of the plants [22,23]. The *δ*^13^C and *δ*^2^H (^2^H/^1^H) of n-C29 alkanes were significantly more positive in olive oils from the southern compared with northern Mediterranean countries [24]. As for Italian EVOOs, works carried on *δ*^13^C and *δ*^18^O [25,26], also in combination with *δ*^2^H [27,28], proved that it wass possible to distinguish samples from different Italian macro areas, as well as Italian from other Mediterranean olive oils. The three isotopic ratios, in particular *δ*^2^H and *δ*^18^O, were found to be correlated to the climatic (mainly temperature) and geographical (mainly latitude and distance from the coast) characteristics and to the *δ*^18^O and *δ*^2^H of the surface waters as well to the year of production [25,26,27,28]. The *δ*^2^H values significantly distinguished olive oils produced on the Adriatic from those from the Tyrrhenian coast of Italy in each year [29]. The combination of isotopic analysis with ^1^H-NMR profiling achieved optimal discrimination between Greece, Spain, Italy, Turkey, Crete, France, and between Italy and Tunisia, the country from which the largest amount of olive oil is imported in Europe [30]. In this study, it was assumed that IRMS would be able to provide relevant information on the three isotopic ratios in the two investigated EVOO classes that could be useful for the confirmation of geographical origin declared on their labels, and possibly for their differentiation.

The aim of this study was dual. In the first part, sensitive analytical methods based on complementary LC-MS and NMR techniques were applied to detect less known chemical markers among various lipid species able to differentiate Italian monocultivar PDO EVOOs obtained on family farms from those purchased in supermarkets. The second goal was to verify the declared geographical origin of the EVOOs from the both classes by IRMS analysis. It was considered that such findings would significantly contribute to EVOO diversification on the market, and would help to clarify the interrelationship between EVOO origin, quality, and price, and in this way support the growth of the niche in the market segment of consumers informed and interested in healthy, quality products with remarkable diversity and clear identity.

## 2. Results and Discussion

### 2.1. LC-ESI-MS/MS Analysis of Free Fatty Acids, Fatty Acid Methyl and Ethyl Esters, Monoglicerides and Triterpenoids

Validation parameters for the method of determination of minor lipid compounds in EVOO by LC-ESI-MS/MS are shown in Tables S3 and S4. All the calibration curves exhibited good linearity (*r^2^* values from 0.95 to 1.00). Limits of quantification ranged from 0.2 to 40 μg/L depending on the compound. The linearity data were used to assess the percentage of matrix effect (% ME), which was reported in Table S3. The matrix effect was found to be insignificant because the obtained variability was close to %RSD repeatability values [31]. Thus, curves prepared in solvent were selected for the quantification. The coefficients of variation (CV%) did not exceed 15% for intra-day assay and 20% for inter-day assay. The average recovery was in the range from 65% to 125% with %RSD less or equal 20%, which was considered satisfactory. The recovery values obtained surpassed 90% for 14 compounds, were between 80% and 90% for 4 compounds and between 70% and 80% for 3 compounds, with %RSD values between 1% and 11%. This indicated good accuracy, recovery, and precision of the method. It is worth mentioning that several lipids for which the method was also validated (Table S1), such as carnitines, glycerophospholipids, and sphingolipids, were not detected in the samples of this study, since their concentrations were below the determined limits of detection (LOD).

In all the investigated EVOO samples 20 minor lipids were identified, including 10 free fatty acids (FFAs), six FFA methyl and ethyl esters, two monoglycerides (MAGs), and two triterpenoids (Table 1). The most abundant among FAAs was oleic (C18:1) followed by palmitic (C16:0), linoleic (C18:2), stearic (C18:0), and palmitoleic (C16:1) acids, which corresponded to the natural distribution of total FAs (esterified in TAGs + FFA) in EVOO in general [6]. The methyl and ethyl esters of the most abundant FFAs, that is oleates, palmitates, and linoleates, dominated the alkyl ester composition, while oleic and linoleic acids were a structural part of the only two identified MAGs (Table 1).

FFAs in olive oil derive from the breakdown of TAGs by lipolysis. There are many factors which can affect the degree of TAG lipolysis, including anomalies during biosynthesis, microbial activity, and environmental factors. Infestation by the olive fly (*Bactrocera oleae*) is a major cause of high FFA content in olives. Damaged olive fruits, delayed fruit processing, and storage in inappropriate conditions result in increased lipolysis rates, while olive oil extraction which is not properly conducted (e.g., prolonged contact between oil and vegetation water) may also result in high FFA values [6,32]. Therefore, the content of FFA is directly related to the quality of olive oil and reflects the care taken from blossoming and fruit set to the eventual sale and consumption of the oil [33]. The FFA content, also known as acidity, is one of the main criteria used to establish different categories of olive oil: according to the European community, EVOO, as the highest quality category, must have FFA content below or equal to 0.8% (as oleic acid, *w*/*w*), as obtained by the standard titration method [2]. In this work, the average total FFA concentration obtained by LC-ESI-MS/MS analysis did not exceed 0.2% in neither of the two investigated EVOO classes (Table 1). Although a tendency towards higher concentrations in commercially-blended than in PDO EVOO was noted for the majority of FFAs, statistically significant differences were found only for linolenic acid. Such a result was, to some extent, in accordance with a previous study in which low-priced EVOO samples were found to contain more FFAs than EVOOs of higher price [34]. FFA content was previously shown to increase during olive oil storage and aging [35,36]. In this work, in contrast to the monocultivar PDO EVOOs which were analyzed relatively fresh, the age of commercially-blended EVOOs was not declared by the producers/sellers and it was practically unknown. It was possible that the samples from the latter class were fully or partially composed from oils obtained in harvests prior to 2016, and that the increased concentrations of particular FFAs partially resulted from TAG chemical hydrolysis during aging. Despite the possible differences with respect to the EVOO age, it must be kept in mind that all the EVOOs included in this study were carefully selected and sampled at the same time, and were therefore valid and authentic representatives of the both classes of EVOOs offered on the market at that given moment.

The content of fatty acid alkyl esters (FAAEs) was principally introduced among the chemical parameters controlled in olive oil quality evaluation [2] to detect blends including low quality olive oils with weak organoleptic defects [17,18]. FAAEs are formed by esterification of short-chain alcohols methanol and ethanol with FAAs yielding methyl and ethyl esters, respectively, although transesterification with triglycerides or partial glycerides may also be a source [37]. They are generally considered indicators of lower olive oil quality and their high concentration often indicate the use of olive fruits with fermentative alterations [17,38,39,40]. In fact, it was demonstrated that FAAE formation was not limited mainly by the content of FAA, but it appeared to be strongly related to the concentration of free alcohols in oil, among which ethanol can be produced exclusively by fermentation [37]. However, evidence exists that the content of FAAE can be relatively high even in high quality EVOO, and vice versa [41]. In this study, the average total concentration of fatty acid ethyl esters (FAEEs) and the total FAAE concentration were below the maximum limit of 35 mg/kg prescribed by the European Commission regulation for EVOO [2]. Higher concentrations of all the identified FAAE/FAEEs were found in commercially-blended EVOOs (Table 1), which indicates the possibility that the olives used for the production of particular samples from this class were overripe or of lower quality suffering from fermentative alterations. Such results are in agreement with our previous report generated from a study with the same sample set, where commercially-blended olive oils were characterized by lower sensory quality on the average, with a number of samples having a sensory defect, including fusty/muddy sediment, vinegary/winey, or musty [4] which could have originated from undesirable fermentative processes, as reported earlier [38,42]. FAAE concentration was previously shown to increase during storage [41,43], which is another possible cause of the higher concentration found in commercially-blended EVOOs, which were possibly not fresh at the moment of sampling.

Similar to diglycerides (DAGs), the presence of MAGs in olive oil is a result of either incomplete biosynthesis of TAGs or their later hydrolysis during processing and storage [12,44]. In virgin olive oil, DAGs are present in the range of 1–2.8%, while MAGs are found in amounts lower than 0.25%, with a much higher proportion of those containing a single fatty acid on position 1 than on position 2 of glycerol moiety [45,46]. The identification of only two 1-MAG species in this work confirmed this phenomenon (Table 1). Both compounds, 1-oleoyl- and 1-linoleoyl-rac-glycerol were found in higher concentration in commercial–blended EVOO, confirming the possibility that a higher degree of TAG hydrolysis occurred in these than in PDO EVOOs.

The largest portion of triterpenoids in olive fruit is located in its epicarp. Triterpenoid concentration in olive oil can be increased by longer malaxation durations and higher malaxation temperatures during olive processing, depending on the compound and olive cultivar [47]. High levels of particular triterpenoids may indicate the presence of olive pomace oil in EVOO, and the percentage of the sum of triterpene diols uvaol and erythrodiol with respect to total sterols is in fact included among the criteria which are evaluated in testing EVOO authenticity in EU [2]. Higher concentrations of the triterpenoids identified in this study, oleanolic acid and uvaol, were found in commercially-blended EVOO (Table 1). Although a possibility should not be neglected that the malaxation and processing parameters were the cause, it must be kept in mind that the content of triterpenoids strongly depends on cultivar origin [47,48], which is therefore another possible source of the observed difference.

### 2.2. LC-ESI-IT-MS Analysis of Triglycerides

Relative proportions (%) of triglycerides (TAGs) obtained by LC-ESI-IT-MS analysis in monocultivar PDO and commercially-blended EVOO are reported in Table 2. As expected, TAGs consisting of the most abundant naturally occurring FAs in olive oil, the species 54:3, 52:2, 54:4, and 52:3, dominated the profiles in both classes of EVOO. The obtained profiles did not fully coincide with those obtainable by the official EU method [2], with some TAGs not reported and some additionally identified, which suggests the method applied in this study could be used as a complementary approach to the standard one for obtaining additional information.

The differences between the two EVOO classes with respect to average TAG composition were not large (Table 2). TAG composition is not evaluated among the parameters related to EVOO sensory quality, while a part of EVOO nutritional value linked to TAGs, related mainly to the FA unsaturation level, depends mostly on cultivar, geographical origin and fruit ripening degree [9,10,11,34]. The differences between the two classes of EVOO, observed for a relatively small number of TAG species, could be primarily ascribed to the abovementioned factors. TAGs 52:3 and 52:6 occurred in a higher average percentage in monocultivar PDO EVOO, while TAGs 54:1 and 54:2 stood out with higher values in commercially-blended EVOO (Table 2). The proportions of the TAG species that could possibly be associated with particular alterations in production with possible repercussions on olive oil quality, such as species that include FAs with odd number of carbon atoms (53:2 and 53:3) and TAG peroxides, did not differ between the two classes.

The proportions of particular minor TAG isomers, not included in the official methods [2] and investigated rather scarcely in olive oil up to date, are reported in Table 3. Significant differences between the two EVOO classes were found only for the relative proportions of TAG 50:4 I and II species. Although the source of the observed differences remained unexplained at this stage, and it could only be assumed that factors such as cultivar and geographical origin, respectively, could have had a significant effect, the results obtained are certainly intriguing and imply the need to further investigate the significance of the trace TAG species in olive oil.

### 2.3. H-NMR Analysis of Lipids

**Figure 1:**
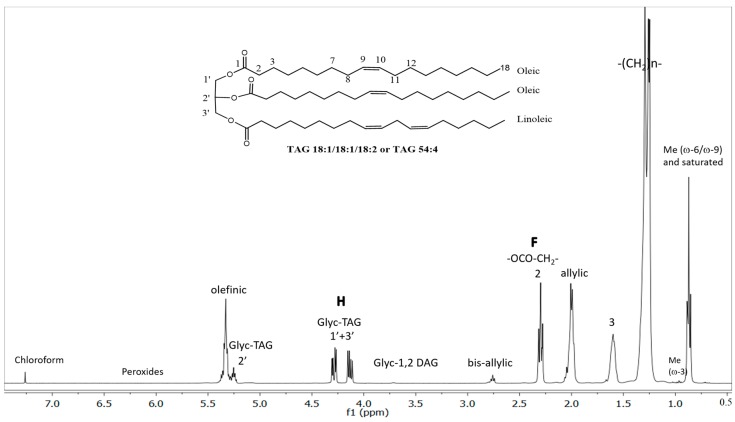
400 MHz proton nuclear magnetic resonance (^1^H-NMR) spectrum of a monocultivar protected designation of origin (PDO) extra virgin olive oil sample in CDCl_3_ at 300 K.

The lipid (essentially TAG) composition of EVOO samples was established by measurement and analysis of the corresponding ^1^H-NMR spectra as described in Section 3.4. The main features of the spectra are outlined in Figure 1 reporting also one of the main TAG species present in olive oils (TAG 54:4, 18:1/18:1/18:2) as a model. The data reported in Table 4 were obtained by area peak integration and simple equations relating them to the number of protons at a given position. 

In particular, the ratio of the peaks area F/H represents the best way to validate the approximations used in the approach for the analysis of edible oils relying on the large dominance of TAG species [49]. Its value should be exactly 1.500 in an oil containing only TAG species, since the signal F (δ_H_ ≈ 2.30 brt, -CH_2_ in α-position in the acyl chains) represents six protons and the signal H (δ_H_ ≈ 4.29 dd and δ_H_ ≈ 4.14 dd, -CH_2_ from sn-1,3 TAG) represents four protons. F/H values higher than 1.500 can be explained by the presence of DAG, MAG, and/or FFA which give their contribution to F but do not contribute to the H peak area. As much as the F/H ratio diverges from 1.500 the contribution of DAG, MAG, and FFA becomes higher, but within the range 1.450 ≤ F/H ≤ 1.550 the approach is still considered reliable. Thus, the F/H ratio indicates whether the approximations are correct and therefore produce trustworthy analytical data for the % molar fraction of SFA, MUFA, and PUFA fatty acyl chains in a targeted olive oil. In this work, the average F/H value of all the analyzed EVOO was 1.512 with a very small relative standard deviation of 0.5%, confirming the calculations were valid and reliable.

A certain degree of intra-class heterogeneity was observed within both classes of investigated EVOO with respect to the relative proportions of total SFA, MUFA, and PUFA. Concerning the relative amount of the lipid acyl chains, a bimodal distribution of PDO EVOOs was noted, with a major set (14 of 20 PDO samples) showing a classical distribution of SFA/MUFA/PUFA = 15.1 ± 1.5/76.3 ± 1.4/8.6 ± 0.9, whilst the remaining lead to the averaged distribution values lower in MUFA, such as SFA/MUFA/PUFA = 16.1 ± 0.8/72.6 ± 0.8/10.4 ± 0.5. Worth of note, particularly unexpected distributions were observed in particular samples, such as in Ottobratico cultivar PDO EVOO from Reggio Calabria with SFA/MUFA/PUFA = 19.0 ± 0.3/70.8 ± 0.3/10.2 ± 0.2 (richer in SFA and PUFA), and in Taggiasca cultivar PDO EVOO from Imperia with SFA/MUFA/PUFA = 12.5 ± 0.1/80.0 ± 0.2/7.5 ± 0.1 (richer in MUFA). For commercially-blended EVOO a similar intra-class differentiation was observed, with the most populated set (19 of 25 samples) centered at average molar fractions SFA/MUFA/PUFA = 15.0 ± 0.9/76.9 ± 1.9/8.1 ± 15 and a minor set (6 of 25) centered at average molar fractions SFA/MUFA/PUFA = 17.1 ± 0.5/72.1 ± 1.2/10.8 ± 1.5. Although the averaged UI (0.941 in the first set versus 0.950 in the second set) and iodine value (84.0 versus 84.5, respectively) of the two mentioned sets of commercially-blended EVOO were quite similar, the second set was characterized by significantly higher relative amounts of SFA and PUFA and lower amount of MUFA (oleic chain, essentially). 

The differences between the two classes of the investigated EVOO with respect to the ^1^H-NMR data can be seen in Table 4. Both classes were characterized by relatively similar major lipid parameters. Monocultivar EVOOs were distinguished by a higher level of linolenic acid and lower iodine value (IV), while a significant difference for unsaturation index (UI) was not found, meaning an unambiguous general conclusion about the difference between the two classes of EVOO with respect to the level of unsaturation could not be made at this point. Level of saturation of acyl chains in olive oil TAGs may depend on various factors, including geographical position and climate, as well as varietal origin [50]. It is possible that these were among the main sources of both intra- and inter-class variability of lipid composition observed in this study. 

Stereospecific distribution of FAs in DAG is known to be affected by several factors. 1,2-DAG isomers are commonly attributed to the incomplete biosynthesis of TAGs in olive fruit, whereas 1,3-DAGs are considered to derive mainly from enzymatic or chemical hydrolysis of TAGs before or during oil extraction [12]. It was shown that 1,2-, 1,3-, and total DAG concentrations in olive oil significantly increase as a result of alterations during processing, including, for example, prolonged storage of piled olives before processing [12,51]. During storage 1,2- species isomerize to more stable 1,3-DAGs, making the ratio of 1,3-/1,2-DAG a useful criterion indicative of olive oil age [12,44,51]. In this study, a higher proportion of 1,2-DAG fraction was found in commercially-blended than in PDO EVOO (Table 4), while 1,3-DAG isomers were not identified. Considering the contents of the other tentative indicators of olive oil age evaluated in this study, such as FFAs and FAAEs, it was tentatively assumed that commercially-blended EVOO were at least partially composed of olive oils obtained during harvests prior to 2016, meaning their age was older, on the average, than that of the PDO ones. Knowing that the concentration of 1,2-DAGs decreases during storage, it was expected that the presumably older commercially-blended EVOO would be characterized by lower amounts in relation to PDO EVOO, but this was not the case. Nevertheless, the possibility cannot be rejected that the commercially-blended samples contained higher average concentration of 1,2-DAGs already at the moment of production and/or release on the market, which later decreased but were still higher than that found in monocultivar PDO EVOOs. 

The differences observed between the average estimated levels of FAA and other minor lipids found in the two classes of EVOO (Table 4) correspond well to those determined for particular FAAs, FAAEs, and MAGs by the LC-ESI-MS/MS method (Table 1). Commercially-blended EVOOs were characterized by higher levels, which was possibly mainly a result of an increased degree of TAG lipolysis, although the contribution of other factors, such as different cultivars and geographical origin, as well as EVOO age and storage conditions, should not be completely excluded.

### 2.4. Multivariate Statistical Analysis

**Figure 2:**
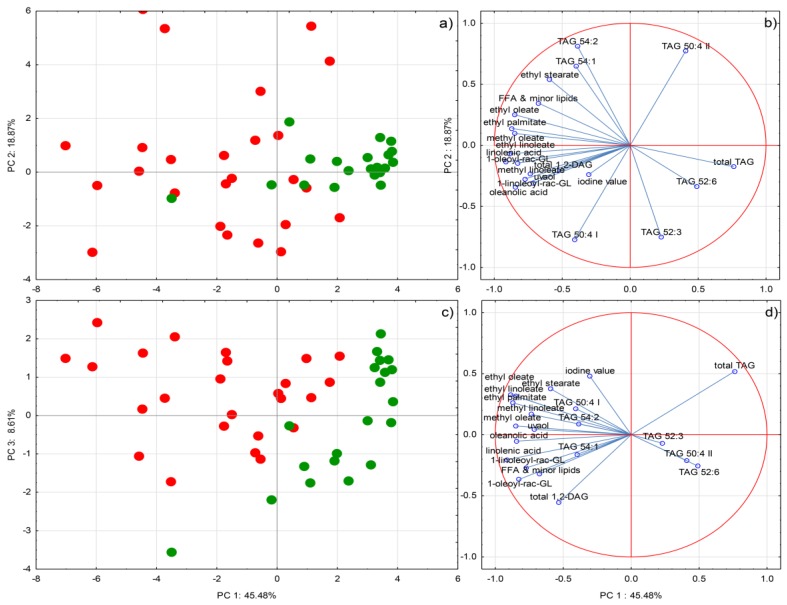
(**a**) and (**c**) Separation of olive oils sold as extra virgin olive oil (EVOO) in Italy according to the origin of purchase in two-dimensional space defined by the first three principal components, PC1, PC2, and PC3. Green cycles represent monocultivar protected designation of origin (PDO)EVOO purchased on family farms, while red cycles represent commercially-blended EVOO purchased in supermarkets (**b**) and (**d**) Factor loadings of selected variables, i.e., concentrations or proportions of various lipid species, obtained by LC-ESI-MS/MS, LC-ESI-IT-MS, and ^1^H-NMR, respectively, on PC1, PC2, and PC3.

Principal component analysis (PCA) separated the samples belonging to the two classes of investigated EVOO according to the origin of purchase relatively successfully (Figure 2). Although monocultivar PDO EVOOs were produced from different olive cultivars grown in different geographical areas in Italy, they were grouped much closer to each other than the commercially-blended ones, suggesting a greater level of intra-class homogeneity with respect to the profile of lipids. It is possible that the presumed differences between the average age of the EVOOs from the two classes, and still a relatively homogenous geographical origin of Italian PDO in comparison to possibly heterogeneous provenience of the commercially-blended EVOOs (Italy and other EU countries), were among the causes. Several markers were found to be related to the commercially-blended EVOOs, including all the minor lipid species, such as FFAs, FAAEs, MAGs, and DAGs, and also particular TAG species and iodine value, which corresponded completely to the one-way ANOVA results. As mentioned above, the positions of the commercially-blended EVOO samples on Cartesian plane were rather dispersed, pointing to the very heterogeneous composition of minor lipids and potential quality of these samples. 

**Figure 3:**
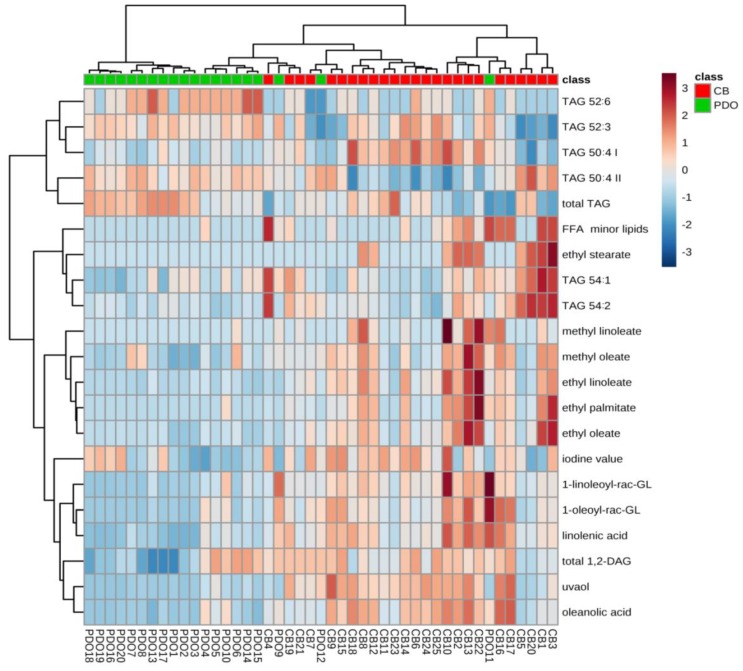
Hierarchical clustering analysis performed using lipid profiles found in Italian monocultivar protected designation of origin (PDO) extra virgin olive oils (EVOO) purchased on family farms and commercially-blended (CB) EVOO purchased in supermarkets in Italy. The heatmap was generated using 21 most significant compounds (the highest Fisher ratios). The rows in the heatmap represent lipids and the columns indicate samples. The colors of the heatmap cells indicate the abundance of lipids across different samples. The color gradient, ranging from dark blue through white to dark red, represents low, middle, and high abundance of lipid species.

Relatively good separation obtained by hierarchical clustering analysis confirmed that the two investigated classes of EVOO differed notably with respect to the composition of lipids (Figure 3). Most of the conclusions were similar to those obtained by the PCA analysis: PDO EVOOs formed a more heterogeneous class, characterized by a smaller number of markers, while the commercially-blended EVOOs exhibited rather diverse lipid composition.

**Figure 4:**
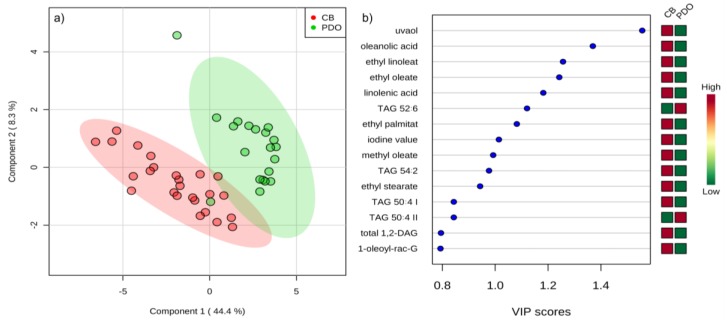
(**a**) Separation of olive oils sold as extra virgin olive oil (EVOO) in Italy according to the origin of purchase in two-dimensional space by partial least squares discriminant analysis. Green cycles represent monocultivar protected designation of origin (PDO) EVOO purchased on family farms, while red cycles represent commercially-blended (CB) EVOO purchased in supermarkets (**b**) variable importance in projection (VIP) scores of the variables (lipids) most useful for the differentiation of monocultivar PDO EVOO and commercially-blended EVOO.

PLSDA allowed a rather good differentiation of the two classes of investigated EVOO according to the origin of purchase (Figure 4). Interestingly, the highest variable importance in projection (VIP) scores were attributed to the triterpenoids, such as uvaol and oleanolic acid, which turned out to be the most important differentiators. Such a result confirmed once again the potential of the compounds from the olive oil unsaponifiable fraction to serve as markers according to various criteria. Besides triterpenoids, the 15 most important lipids according to PLSDA included particular FAAE and other minor lipid species abundant in commercially-blended EVOO, while certain TAGs were confirmed as related to monocultivar PDO EVOO.

### 2.5. Confirmation of Geographical Origin by Isotope Ratio Mass Spectrometry (IRMS)

The average values of δ^13^C, δ^2^H, and δ^18^O in monocultivar PDO and commercially-blended EVOO are reported in Table 5. Statistically significant differences were determined for δ^2^H and δ^18^O, with lower values found in monocultivar PDO EVOO. According to the literature [30], the isotopic values of olive oil increase with decreasing latitude. It is possible that the contribution of Italian monocultivar PDO EVOO originating from the orchards located further from the sea and at higher latitudes with colder climate (e.g., Brescia, Verona, and Garda PDOs) prevailed and significantly decreased the average δ^2^H and δ^18^O isotopic values in monocultivar PDO EVOO, the same as non-Italian commercial EVOO originating from lower latitudes in warmer EU Mediterranean countries possibly had a notable influence on increasing the average δ^2^H and δ^18^O values determined in commercially-blended EVOO.

**Figure 5:**
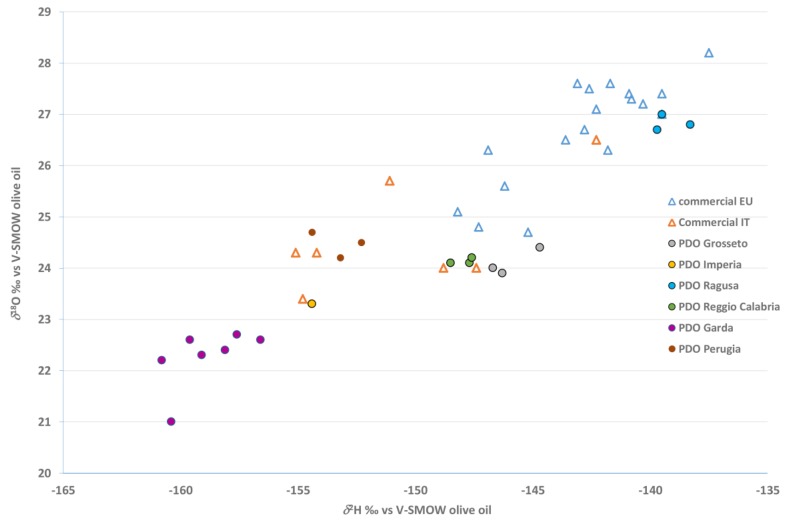
Differentiation of Italian monocultivar protected designation of origin (PDO) extra virgin olive oils purchased on family farms and commercially-blended extra virgin olive oils (EU and Italian origin) purchased in supermarkets in Italy according to *δ*^2^H and *δ*^18^O stable isotopic ratios.

By correlating the two parameters more linked to geographical origin, i.e., δ^2^H and δ^18^O, it was possible to visualize different groupings (Figure 5). As already observed [27], Garda PDO EVOO (Casaliva cultivar) was characterized by the lowest δ^2^H and δ^18^O values, most probably due to the production area (far from the sea, higher latitude) and climate (colder than the Mediterranean one). The commercial non-Italian EU EVOO showed the highest δ^2^H and δ^18^O values, overlapping only with those of Ragusa PDO (Tonda Iblea cultivar), because the geographical and climatic characteristics of Sicily are similar to those of other European Mediterranean countries, as observed previously [30]. The commercially-blended EVOOs of Italian origin had δ^2^H and δ^18^O values overlapping with those of Italian monocultivar PDO with the exception of Garda PDO EVOO, which was as expected because the areas of production overlapped as well. 

## 3. Materials and Methods

### 3.1. EVOO Samples

After preliminary selection from a larger group of high quality monocultivar EVOOs with PDO, samples that were produced from olives of Italian cultivars harvested in 2016 were collected from different geographical areas in Italy (price range from 20 to 30 €/L), including Reggio Calabria (cultivar: Ottobratica; *n* = 3), Perugia (cultivar: Moraiolo; *n* = 3), Ragusa (cultivar: Tonda Iblea; *n* = 3), Grosseto (cultivar: Frantoio; *n* = 3), Imperia (cultivar: Taggiasca; *n* = 1), Brescia (Garda Bresciano PDO, cultivar: Moraiolo; *n* = 1), Verona (Garda Orientale PDO, cultivar: Leccino; *n* = 1), and Riva del Garda (Garda Trentino PDO, cultivar: Casaliva; *n* = 5). Furthermore, 25 commercially-blended EVOOs were selected according to Nielsen data (New York, NY, USA 2016) as among the most consumed during 2016 in Italy (price range from 3 to 12 €/L) and were purchased from Italian grocery stores (supermarkets), consisting of seven samples with Italian and 18 samples with EU origin declared on their labels. All the samples were stored in dark glass bottles at a controlled temperature of 15 °C before analysis, and gaseous N_2_ was added in the headspace to prevent oxidation each time the bottles were opened.

### 3.2. Standards and Solvents 

The solvents used for the analysis of lipids in EVOO were LC-MS grade methanol, hexane, isopropanol and formic acid, purchased from Honeywell Riedel-de Haën (Seelze, Germany) and all aqueous solutions, including the HPLC mobile phase, were prepared with water purified using a Milli-Q system (Millipore, Vimodrone, Milan, Italy). All the analytical standards used for identification and calibration are listed in Table S1.

### 3.3. LC-ESI-MS/MS Analysis

The samples were prepared by weighing 500 mg of oil in a 10 mL flask, brought to volume with a 2-propanol solution and internal standard (stearic acid d3 at 1 mg/L). The final solutions were filtered through 0.22 μm filters and transferred into 2 mL vials [52]. LC-ESI-MS/MS analysis of FFA and other lipids was carried out using a UHPLC Dionex 3000 (Thermo Fisher Scientific, Dreieich, Germany), coupled to an API 5500 triple-quadrupole mass spectrometer (Applied Biosystems/MDS Sciex, Toronto, ON, Canada) equipped with an electrospray source. Five microliters of sample were injected into the LC-ESI-MS/MS system using an autosampler (Dionex Thermo Fisher Scientific, Germany) kept at 10 °C. A reversed phase column Ascentis Express C18 (150 mm × 2.1 mm, 2.7 μm; Sigma, Milan, Italy) set at 55 °C was used for the compound separation. Flow-rate was 0.26 mL/min and the composition of mobile phases was: solvent A (CH_3_CN 40% in water, NH_4_COOH 10 mM and HCOOH 0.1%) and solvent B (CH_3_CH(OH)CH_3_ 90%, CH_3_CN 10%, NH_4_COOH 10 mM and HCOOH 0.1%). Separation was carried out following a 30 min multistep linear gradient, according to the method reported by Della Corte et al. [53]. Selected chemical standards were used to construct calibration curves and data were expressed as mg/kg after normalization on the basis of the internal standard stearic acid d3. The targeted lipids were detected under multiple reaction monitoring (MRM) mode and the compounds were identified based on their reference standards, retention times, and qualifier and quantifier ions (Table S2). The chromatographic system and data acquisition were managed by Analyst™ software version 1.6.1 (Applera Corporation, Norwalk, CT, USA).

For the method validation, the US Food and Drug Administration (FDA) recommendations for a bioanalytical method were followed [54]. The validation included the evaluation of linearity, sensitivity, variability, recovery, accuracy, and precision based on calibration standards and quality control (QC). Calibration curves were made in 2-propanol and lipid matrix [53,55] in order to evaluate the percentage of matrix effect (%ME) for each compound. The values were determined by comparing the equality in the slope ratio between the curves in solvent and matrix, using the following formula: %ME = 100% × (1 − slope solvent/slope matrix) [56]. The regression line was created with the least square fit, and the determination coefficient (*r^2^*) was also calculated. The linearity was evaluated by preparing different levels of independent calibration and adding increasing concentrations of each lipid in different concentration ranges. The sensitivity of the method was evaluated with limits of detection (LOD) and limits of quantification (LOQ) at the concentration in which the quantizer transitions showed a signal to noise ratio (S/N) of >3 and >10, respectively. To estimate the analytical variability, intra-day and inter-day parameters were calculated by injecting 10 times a middle concentration level QC sample on the same day and re-injecting it for six consecutive days. Intra-day and inter-day variability were evaluated by the coefficients of variation (CV%). The recovery test was carried out to verify the applicability of the LC-ESI-MS/MS technique, and it was determined as the average of the “measured value”/“the expected value” ratio (%). The precision values were calculated as relative standard deviation (%RSD) among the measures replicated in the QC sample. They were obtained by analyzing the same sample 10 times. Since the precision can vary with the concentration, it was appropriated to analyze at least three samples at different concentrations (low, medium, and high) with respect to the calibration range for each analyte. The accuracy was calculated as the difference between the calculated value and the theoretical value divided by the theoretical value, reported as the relative error percentage (%RE).

### 3.4. H-NMR Analysis of Lipids

All the EVOO samples were prepared by addition and suitable mixing of 700 μL of deuterated solvent (CDCl_3_) to 200 μL of oil (solution about 200 mM) in a 5 mm NMR tube. All the ^1^H-NMR spectra were acquired at 300 K on a Bruker-Avance 400 MHz NMR spectrometer (Bruker, Bremen, Germany) by using a 5 mm BBI probe with 60° hard pulse length of 6.6 µs at a transmission power of 0 db. For the acquisition, 32 K complex points were recorded, the spectral width was set to 10 ppm, the frequency offset was set to 4.8 ppm, the relaxation delay was set to 15 s, the acquisition time was 8.2 s, the number of scans was set to 32, and the number of dummy scans was equal to 2. The total experimental time was 13 min. All spectra were acquired without spinning. The chemical shift scale was calibrated by using the residual proton signal of the deuterated solvent (CHCl_3_ signal at 7.260 ppm). The data were acquired using the software Topspin 2.1 (Bruker Biospin, Rheinstetten, Germany). The resulting spectra were processed manually and automatically with the software MestreNova 12.0.0 (Mestrelab Research SL, Santiago de Compostela, Spain) taking care to achieve good symmetry on all peaks. The baseline was corrected using a polynomial function. The integral data extracted from the spectra were analyzed using standard software (Microsoft Office Excel 2016). The ratio of the peaks area F/H (Figure 1) was tested to validate the approximations used in this study for calcuation of various lipid species, as suggested earlier [49]. The average F/H value of all the EVOO analyzed in this work was 1.512 with a very small relative standard deviation of 0.5%, confirming the approximations of the calculations were fulfilled. The data reported were obtained by the following procedures. The % molar fraction of α-linolenic acid (18:3) was obtained by the ratio of the peak area of ω-3 Me (δ_H_ = 0.97 t) with respect to the peak area of F (×2/3), the % molar fraction of linoleic acid (18:2) by the ratio of the peak area of bis-allylic protons (δ_H_ = 2.77 brt) with respect to the peak area of F minus the contribution of the previously evaluated % molar fraction of α-linolenic acid (i.e., %18:2 = %PUFA − %18:3). Finally, MUFA (16:1 + 18:1, essentially) was evaluated by the peak area of allylic protons (δ_H_ = 2.01 brt) with respect to the peak area of F minus the contribution of the previously evaluated % molar fraction of PUFA, whilst SFA (16:0 + 18:0 essentially) was evaluated from total peak area of methyl protons (δ_H_ = 0.97 + δ_H_ = 0.89) with respect to the peak area of F (×2/3) minus the contribution of PUFA and MUFA. The relative contribution of 1,2-DAG was given by the integration of the peak at δ_H_ = 3.71 brd attributable to the -CH_2_ from sn-1,2-DAG, always present in minor amount in olive oils. From these data the average unsaturation index (UI) and the iodine value (IV) of the acyl chains in TAG were evaluated. Worth of note, the standard deviations of all the mentioned measurements, calculated from three technical replicates (ex novo acquisition and data analysis) of a given EVOO sample, were quite low (<1%) for SFA, MUFA, linoleic and linolenic fatty acyl chains, giving good reliability to the approach applied in this study.

### 3.5. LC-ESI-IT-MS Analysis of Triglycerides

LC-ESI-IT-MS analysis of the olive oils was performed on a Hewlett-Packard Model 1100 Series liquid chromatograph coupled both to an Agilent 1100 Series DAD (Photo Diode-Array Detector) (Hewlett–Packard Development Company, L.P., Palo Alto, CA, USA), and to a Bruker Esquire-LC quadrupole ion-trap mass spectrometer equipped with atmospheric pressure ESI+ interface (ESI-IT-MS). Isocratic elution was applied with a flow of 0.3 mL/min by using a Kinetex C_18_ column (2.6 μm 100A 100 × 2.1 mm) (Phenomenex, Sydney, Australia) as stationary phase, and isopropanol:methanol 10/90 (*v*/*v*), 10 mM in ammonium acetate, as mobile phase. Samples were prepared by diluting 1:200 (5 mM) each EVOO with a solution of MeOH:CHCl_3_ 8/2 (*v*/*v*), and 4 μL were injected. Relative quantitation of TAG species was established by peak area integration of the MS extracted ion currents of the corresponding major ions produced by ESI ionization by assuming the same response time for all the TAGs species.

### 3.6. IRMS Analysis

The analysis of the stable isotope ratios of H, C and O was performed on the bulk olive oil. ^13^C/^12^C (δ^13^C) was measured (around 0.5 mg of oil) using an isotope ratio mass spectrometer IsoPrime (Isoprime Limited, Manchester, UK) following total combustion in an elemental analyzer (VARIO CUBE, Elementar, Hanau, Germany). ^18^O/^16^O (δ^18^O) and ^2^H/^1^H (δ^2^H) were measured (around 0.3 mg of oil) using an IRMS (Finnigan DELTA XP, Thermo Scientific, Bremen, Germany) coupled with a pyrolyzer (Finnigan TC/EA, high temperature conversion elemental analyzer, Thermo Scientific). For δ^2^H and δ^18^O analysis, the weighed samples were stored in a desiccator above P_2_O_5_ for at least four days before analysis, then put into an auto-sampler equipped with a suitable cover. During measurement, dryness was guaranteed by flushing nitrogen continuously over the samples. Before determining the δ^2^Η values, the H3+ factor was verified to be lower than 8, as suggested in the instrumental manual.

The values were denoted in delta in relation to the international V-PDB (Vienna-Pee Dee Belemnite) for δ^13^C and Vienna-standard mean ocean water (V-SMOW) for δ^18^O and δ^2^H, according to the following general equation: δi E = (i RSA − i RREF), where i is the mass number of the heavier isotope of element E, RSA is the respective isotope ratio of the sample and RREF is the relevant internationally recognized reference material [57]. The delta values were multiplied by 1000 and expressed in units “per mil” (‰). The δ^13^C and δ^2^Η values were calculated against two international reference materials (Icosanoic Acid Methyl Esters USGS70, δ^13^C value: −30.53‰ and δ^2^H value: −183.9‰ and USGS71, δ^13^C value: −10.5‰ and δ^2^H value: −4.9‰), through the creation of a linear equation.

δ^18^O was calculated against IAEA 601 (benzoic acid δ^18^O = +23.3‰) and 602 (benzoic acid δ^18^O = +71.4‰), through the creation of a linear equation. Data were therefore reported relative to V-PDB on a scale normalized to LSVEC-NBS19 for δ^13^C, and relative to the V-SMOW-SLAP scale for δ^2^Η and δ^18^O. The uncertainty (2 s) of measurements, calculated following the Nordtest approach, which combines within-laboratory reproducibility standard deviation and laboratory bias using PT data [58], was < 0.3‰ for δ^13^C analysis, < 0.5‰ for δ^18^O and < 3‰ for δ^2^H.

### 3.7. Statistical Data Elaboration

Data obtained by the LC-ESI-MS/MS analysis of minor lipids, LC-ESI-IT-MS profiling of TAGs, ^1^H-NMR analysis of major lipid parameters, and IRMS analysis in the investigated EVOO were subjected to one-way analysis of variance (ANOVA) and the average values were compared by least significant difference (LSD) test at the level of *p* < 0.05. The data were further processed by principal component analysis (PCA) in order to better visualize the differences between the two classes of EVOO and explain them on the basis of the content and composition of various lipid species. Prior to PCA, the original datasets were reduced to include only the lipids and parameters for which statistically significant difference between the two classes was determined by one-way ANOVA. Partial least squares discriminant analysis (PLSDA) was performed to extract the most useful variables among lipids for the differentiation of the two investigated classes of EVOO: variable importance in projection (VIP) scores for lipids were determined as the weighted sums of the squares of the weight in the PLSDA. Hierarchical clustering was conducted and a heatmap was generated by Ward algorithm and Euclidean distance analysis. Multivariate statistical elaboration was performed on mean-centered data. ANOVA and PCA data elaboration was performed using Statistica v. 13.2 software (StatSoft Inc., Tulsa, OK, USA), while PLSDA and cluster analysis was conducted using MetaboAnalyst v. 4.0 (http://www.metaboanalyst.ca) created at the University of Alberta, Canada [59].

## 4. Conclusions

LC-MS and NMR techniques were found to be potent tools to study the variability of various lipid species in EVOO. Their outputs were shown to be relatively complementary and their combined use successfully extracted several chemical markers useful for the differentiation of the two classes of EVOO with respect to the origin of purchase: monocultivar PDO EVOO from family farms vs. commercially-blended EVOO from supermarkets. Considering that the EVOO samples from both classes were characterized by known (for PDO) and declared/presumed (for commercially-blended) geographical and pedoclimatic heterogeneity and large variations in olive growing and oil producing parameters, the extracted markers could be considered relatively robust. Commercially-blended EVOO contained higher concentrations of the majority of minor lipids, including FAAs, FAAEs, MAGs, and DAGs, which may be indicative of a higher degree of TAG lipolysis in these than in monocultivar PDO EVOO. However, triterpenoids and particular TAG species were also found in higher concentrations/proportions in the samples from the commercially-blended EVOO class, suggesting a possible influence of other factors, including diverse cultivar and geographical origin, respectively. The results of IRMS analysis were reasonably consistent with the information about the geographical origin declared on the labels of the investigated EVOOs, which showed considerable variability. The results of this study undoubtedly confirmed the heterogeneity of oils which are sold declared as EVOO in Italy in terms of their lipid composition and geographical origin. In that sense, the obtained findings could significantly contribute to EVOO diversification on the market, and could help to clarify the interrelationship between EVOO origin, quality, and price, and in this way support the growth of the niche in the market segment of consumers informed and interested in healthy, quality products with remarkable diversity and clear identity.

## Figures and Tables

**Table 1:** Concentrations (µg/g) of minor lipids in monocultivar protected designation of origin (PDO) and commercially-blended extra virgin olive oils obtained by LC-ESI-MS/MS analysis. An asterisk (*) in a row represents significant differences between mean values at *p* < 0.05 obtained by ANOVA and least significant difference (LSD) test.

Minor Lipids	Origin/Class	
	Monocultivar PDO	Commercially-Blended
*Free fatty acids*		
Palmitic acid (C16:0)	285.41	362.95
Palmitoleic acid (C16:1)	10.58	16.57
Stearic acid (C18:0)	27.41	43.39
Oleic acid (C18:1)	809.53	1045.52
Linoleic acid (C18:2)	70.52	93.10
Linolenic acid (C18:3)	5.67	9.76 *
Arachidic acid (C20:0)	8.53	11.42
Behenic acid (C22:0)	8.52	9.45
Erucic acid (C22:1)	0.06	0.05
Lignoceric acid (C24:0)	21.85	27.04
Total free fatty acids	1248.09	1619.27
*Free fatty acid esters*		
Methyl oleate	4.02	7.09 *
Methyl linoleate	0.05	0.24 *
Ethyl palmitate	0.10	1.01 *
Ethyl stearate	0.00	0.32 *
Ethyl oleate	2.29	8.18 *
Ethyl linoleate	0.38	1.04 *
Total free fatty acid esters	6.83	17.87 *
*Monoglycerides*		
1-Oleoyl-rac-glycerol	30.11	53.29 *
1-Linoleoyl-rac-glycerol	8.77	12.84 *
Total monoglycerides	38.88	66.13 *
*Triterpenoids*		
Oleanolic acid	26.71	50.13 *
Uvaol	4.58	14.04 *
Total triterpenoids	31.28	64.17 *

**Table 2:** Relative proportions (%) of triglycerides (TAGs) in monocultivar protected designation of origin (PDO) and commercially-blended extra virgin olive oils obtained by liquid chromatography with quadrupole ion-trap mass spectrometry (LC-ESI-IT-MS) analysis. An asterisk (*) in a row represents significant differences between mean values at *p* < 0.05 obtained by ANOVA and least significant difference (LSD) test.

TAG Species ^†^	TAG Chains ^‡^			Origin/Class	
				Monocultivar PDO	Commercially-Blended
TAG 50:1	18:1	16:0	16:0	5.83	5.44
TAG 50:2	18:1	16:1	16:0	3.06	2.84
TAG 50:3	-	-	-	0.63	0.70
TAG 50:4	-	-	-	0.07	0.08
TAG 52:1	18:1	18:0	16:0	2.32	2.47
TAG 52:2	18:1	18:1	16:0	19.10	19.06
TAG 52:3	18:2	18:1	16:0	10.36 *	9.64
TAG 52:4	18:3	18:1	16:0	3.38	3.13
TAG 52:5	-	-	-	0.46	0.42
TAG 52:6	-	-	-	0.05 *	0.03
TAG 53:2	18:1	18:1	17:0	0.42	0.44
TAG 53:3	18:1	18:1	17:1	0.68	0.70
TAG 53:4	-	-	-	0.12	0.14
TAG 54:1	-	-	-	0.68	0.80 *
TAG 54:2	18:1	18:1	18:0	6.37	7.34 *
TAG 54:3	18:1	18:1	18:1	25.40	26.59
TAG 54:4	18:2	18:1	18:1	11.86	11.56
TAG 54:5	18:3	18:1	18:1	4.55	4.47
TAG 54:6	-	-	-	0.74	0.70
TAG 56:1	-	-	-	0.16	0.18
TAG 56:2	20:0	18:1	18:1	1.47	1.51
TAG 56:3	20:1	18:1	18:1	1.63	1.53
TAG 56:4	-	-	-	0.43	0.38
TAG 58:1	-	-	-	0.06	0.06
TAG 58:2	22:0	18:1	18:1	0.38	0.39
TAG 58:3	-	-	-	0.11	0.10
TAG peroxides ^§^	-	-	-	0.77	0.72

^†^ Number of carbon atoms: double bonds in the structure of the corresponding TAG; ^‡^ number of carbon atoms: double bonds in each of the three fatty acids in the structure of the corresponding TAG; ^§^ proportion (%) of the sum of 52:2, 52:3, 54:3, 54:4, and 54:5 TAG peroxides in total TAGs.

**Table 3:** Relative proportions (%) of minor triglycerides (TAGs) and particular TAG isomers in monocultivar protected designation of origin (PDO) and commercially-blended extra virgin olive oils obtained by HPLC-HRMS analysis. An asterisk (*) in a row represents significant differences between mean values at *p* < 0.05 obtained by ANOVA and least significant difference (LSD) test.

TAG Species ^†^	TAG Chains ^‡^			Origin/Class	
				Monocultivar PDO	Commercially-Blended
*TAG 50:4 isomers*					
TAG 50:4 I	18:2	16:1	16:1	24.54	31.20 *
TAG 50:4 II	18:3	16:1	16:0	75.46 *	68.80
total	-	-	-	100.00	100.00
*TAG 50:3 isomers*					
TAG 50:3 II	18:2	16:1	16:0	66.56	67.55
TAG 50:3 III	18:3	16:0	16:0	33.44	32.45
total	18:1	18:1	16:0	100.00	100.00
*TAG 56:3 isomers*					
TAG 56:3 I	20:1	18:1	18:1	71.63	71.96
TAG 56:3 II	20:0	18:2	18:1	28.37	28.04
total	-	-	-	100.00	100.00
*TAG 53 isomers*					
TAG 53:4	-	-	-	10.12	10.41
TAG 53:3	18:1	18:1	17:1	56.17	54.79
TAG 53:2	18:1	18:1	17:0	33.71	34.80
total	18:1	18:1	18:0	100.00	100.00

^†^ Number of carbon atoms: double bonds in the structure of the corresponding TAG; ^‡^ Number of carbon atoms: double bonds in each of the three fatty acids in the structure of the corresponding TAG.

**Table 4:** Lipids in monocultivar protected designation of origin (PDO) and commercially-blended extra virgin olive oils obtained by NMR analysis. An asterisk (*) in a row represents significant differences between mean values at *p* < 0.05 obtained by ANOVA and least significant difference (LSD) test.

Lipids	Origin/Class	
	Monocultivar PDO	Commercially-Blended
Total triglycerides (TAGs)	99.38	99.05
Total saturated fatty acids (SFA)	15.83	15.52
Total monounsaturated fatty acids (MUFA)	74.95	75.77
Linoleic acid in TAG (C18:2)	7.92	7.61
Linolenic acid in TAG (C18:3)	1.30 *	1.10
Unsaturation index (UI)	0.95	0.94
Iodine value (IV)	82.33	84.10 *
Total 1,2-diacylglycerols (1,2-DAGs)	0.56	0.72 *
Estimated free fatty acids and minor lipids	0.07	0.26 *

**Table 5:** Average values of stable isotopic ratios obtained for monocultivar protected designation of origin (PDO) and commercially-blended extra virgin olive oils obtained by isotope ratio mass spectrometry (IRMS). An asterisk (*) in a row represents significant differences between mean values at *p* < 0.05 obtained by ANOVA and least significant difference (LSD) test.

Stable Isotopic Ratios	Origin/Class	
	Monocultivar PDO	Commercially-Blended
*δ*^13^C	−29.65	−29.44
*δ*^2^H	−151.28	−144.96 *
*δ*^18^O	23.89	26.10 *

## Supplementary Materials

The following are available online, Table S1: standards of minor lipids used in the LC-ESI-MS/MS method development. Table S2: mass transitions (MRM) and instrumental parameters optimized for each metabolite for the analyses by LC-ESI-MS/MS. Table S3: method validation parameters for each metabolite for the analyses by LC-ESI-MS/MS - part I. Table S4: method validation parameters for each metabolite for the analyses by LC-ESI-MS/MS - part II.
